# Diagnostic Differences in Expert Second-Opinion Consultation Cases at a Tertiary Sarcoma Center

**DOI:** 10.1155/2020/9810170

**Published:** 2020-09-29

**Authors:** Asha Rupani, Magnus Hallin, Robin L. Jones, Cyril Fisher, Khin Thway

**Affiliations:** ^1^Sarcoma Unit, Royal Marsden Hospital, London SW3 6JJ, UK; ^2^The Institute of Cancer Research, London SW3 6JB, UK; ^3^Department of Musculoskeletal Pathology, Royal Orthopaedic Hospital NHS Foundation Trust, Robert Aitken Institute for Clinical Research, University of Birmingham, Birmingham B15 2TT, UK

## Abstract

Soft tissue tumors are diagnostically challenging, and it is recommended that these are reported or reviewed by specialist soft tissue pathologists. We present our experience with second-opinion (consultation) cases in a specialist tertiary sarcoma center. The aim of this study was to determine areas of diagnostic difficulty in soft tissue pathology. We assessed 581 second-opinion cases which were reviewed by two experienced pathologists in a period of one year. There was 62% concordance between the original and the second-opinion diagnosis, with diagnostic discrepancy in 38%. The largest group of soft tissue neoplasms received for second opinion was fibroblastic/myofibroblastic tumors, and most major diagnostic problems were encountered in adipocytic and so-called “fibrohistiocytic” tumors. Major diagnostic errors impacting management were found in 148 cases (25%). Morphologic assessment of tumors, judicious use of molecular techniques, newer immunostains and their interpretation, along with importance of knowledge of rarer entities were found to be most useful in avoiding errors.

## 1. Introduction

Soft tissue tumors are diagnostically challenging due to their rarity, swiftly-evolving histopathologic and molecular diagnostic classification, overlapping morphology and immunophenotype with many other neoplasms, and dependence on ancillary immunohistochemical and molecular investigations for many diagnoses. The UK National Institute for Health and Care Excellence (NICE) Guidelines indicate that all soft tissue tumors should be either reported or reviewed by soft tissue pathologists who regularly report them, participate in the UK National Soft Tissue Pathology External Quality Assurance scheme, and are core members of a Sarcoma Multidisciplinary Team [1]. The incidence of malignant soft tissue tumors is around 25–50 per million in the general population [2, 3]. The Sarcoma Unit at the Royal Marsden Hospital, a tertiary, comprehensive cancer treatment center receives a total of approximately 3500 cases of soft tissue neoplasms every year [4, 5], comprising biopsies or resections performed at the center, referral material, and second-opinion/consultation cases. Referral cases are usually previously reported cases from other hospitals, sent when the patient is referred to a soft tissue specialist center for further management or clinical opinion. Second-opinion cases are distinct from referrals as they are usually sought directly by general pathologists for rarer and diagnostically challenging cases. Our center has previously audited diagnostic discrepancy rates and causes for referred cases of soft tissue neoplasms [4, 5], but there are few studies assessing diagnostic discrepancies for second-opinion cases in soft tissue pathology [6–9]. This is important, particularly now in an era where there is widespread routine use of ancillary molecular diagnostic modalities and immunohistochemical assays that can serve as correlates of molecular genetic alterations [10–12]. In this study, we assessed the areas of diagnostic discrepancy in the reporting of soft tissue neoplasms referred for expert opinion, to assess specific discrepancy patterns between general and specialist soft tissue pathologists.

## 2. Materials and Methods

A retrospective audit was performed for second-opinion cases received in the Sarcoma Unit of the Royal Marsden Hospital. Cases were retrieved from the pathology second-opinion database. The cases were received as paraffin blocks, slides, or a combination of both, depending on the local policies of the referring institution, with a covering letter from the referring pathologist and usually the original report. The second opinion was given in the form of a letter addressed to the referring pathologist. Details from each case were obtained from the hospital electronic patient record, or from the hospital pathology document retrieval system to which completed documents are scanned. Original reports or descriptions from the referring pathologists were compared with the second-opinion diagnosis for differences in diagnosis, and, where comparable, other parameters such as grading and use of additional ancillary techniques were recorded. In cases where differential diagnoses were offered by the referring pathologist, the most favoured diagnosis was used for the purpose of analysis. Grading was assigned according to the system by the French Federation of Cancer Centers Sarcoma Group (FNCLCC) [13, 14].

The cases were categorized according to differentiation, and behavior was categorized as malignant, intermediate, or benign based on the World Health Organization classification (2013) of tumors of soft tissue and bone [3]. Minor discrepancies can be those of diagnosis, classification, or grading, where the discrepancy was not thought to provoke significant management change, whereas major discrepancies are defined as those that could instigate significant change in clinical management, resulting in under- or overtreatment. Major discrepancies can be divided into six categories: (1) malignant ⟶ malignant (resulting in significant management change), (2) malignant ⟶ benign, (3) benign ⟶ malignant, (4) mesenchymal ⟶ nonmesenchymal, (5) other (e.g., benign ⟶ benign, but resulting in significant management change), and (6) major grading discrepancies, comprising tumors in which there was any interchange of grade between grades 2-3 and grade 1 (as this could lead to change in management) [4, 5]. The groups in this study were divided into (1) malignant to malignant, (2) malignant to benign, (3) malignant to intermediate, (4) benign to benign, (5) benign to malignant, (6) benign to intermediate, (7) intermediate to intermediate, (8) intermediate to benign, and (9) intermediate to malignant. The reasons for discrepancy were analyzed, and challenging groups of cases are discussed along with some of the diagnostic pitfalls. The results were also compared to the two previous audits from this center addressing referral cases [4, 5].

## 3. Results

A total of 581 cases were received for second opinion in the one-year (2012) period assessed. 360 cases (62%) showed no change in diagnosis and 221 cases (38%) showed a difference between the original diagnosis and the second-opinion diagnosis (summarized in Table 1). Tumors with fibroblastic/myofibroblastic differentiation comprised the largest group of neoplasms received for second opinion (99 cases, 17%). The diagnosis was concordant in 65 cases, with a difference in opinion in 34 cases (34%) (Table 2). Adipocytic tumors comprised the next largest group of soft tissue neoplasms received for second opinion and showed significant discordance between the original and second opinion diagnoses. There were 74 cases, of which 32 (43%) showed diagnostic discrepancy (Table 3). Smooth muscle tumors represented the third largest group with 35 cases, of which discrepancy was seen in 10 (28%) (Table 4). The next group was of vascular tumors with 32 cases, of which discrepancy was noted in 10 (31%) (Table 5). Nerve sheath tumors also comprised 32 cases, with discrepancy in 11 (34%) (Table 6). The next group was of skeletal muscle tumors with 14 cases, with discrepancy in two (10%). A case of benign fibrous histiocytoma was diagnosed as spindle cell rhabdomyosarcoma on morphological appearances. The second case with differential diagnosis of solitary fibrous tumor (SFT) and synovial sarcoma was diagnosed as spindle cell rhabdomyosarcoma on morphology and negative TLE1 immunohistochemistry. The group of so-called “fibrohistiocytic” tumors included 25 cases, of which the diagnosis was discrepant in 16 (64%) (Table 7). In one case with uncertain diagnosis, a second opinion diagnosis of fibrohistiocytic neoplasm of uncertain malignant potential was offered.

Analysis was also performed between the broad categories of malignant, intermediate, and benign soft tissue neoplasms, as change in classification of behavior can have impact on patient management (as summarized in Table 8). 285 cases (49%) were received for second opinion from academic/university teaching hospitals, while 242 (42%) were from community (district general) hospitals, 38 from private clinics, and 16 from overseas university/teaching hospitals. The percentage of concordance of diagnosis based on the type of referring institute is shown in Table 9.

## 4. Discussion

Soft tissue neoplasms represent a heterogeneous group of tumors with a wide range of clinical behaviors. Prognostication and appropriate treatment, including with targeted therapies, is dependent on accurate diagnosis and behavioral stratification. Because of the increasing subspecialization of surgical pathologists, there is a tendency for less frequent exposure of general pathologists to soft tissue neoplasms. As sarcomas comprise only 1% of all adult cancers, even experienced general pathologists see insufficient numbers to gain familiarity or expertise. The error rate in histopathological diagnosis of soft tissue sarcomas in the recent literature is between 14% and 47%, which reiterates the importance of obtaining further opinion from a specialist soft tissue pathologist for these neoplasms [6–9].

Arbiser et al. reviewed 500 cases of soft tissue lesions referred for second opinion. There was diagnostic agreement in 68%, with major discrepancy in 25% and minor discrepancy in 7%, and total diagnostic discrepancy of 32% [6]. Their distribution of discrepant cases was of benign mesenchymal lesions diagnosed as sarcoma in 45%, sarcoma as benign tumors in 23%, nonmesenchymal lesions diagnosed as sarcoma in 20%, and major grading discrepancies in 12%. In our study, we found a discrepancy rate of 38% (Table 1). The first group included cases of spindle cell lipoma and fat necrosis diagnosed as liposarcoma, fasciitis diagnosed as sarcoma, and cellular fibrohistiocytic tumors and tenosynovial giant cell tumor diagnosed as sarcoma. The second group comprised sarcoma (not otherwise specified) (NOS) and leiomyosarcoma misdiagnosed as benign lesions. Nonmesenchymal lesions such as melanoma, carcinoma, and lymphoma were mistaken for sarcoma in the third group, particularly desmoplastic neurotropic melanoma misdiagnosed as malignant peripheral nerve sheath tumor (MPNST). In their experience as well as ours, with the exception of nonmesenchymal lesions, the diagnosis for all major discrepant cases could be made on the basis of the hematoxylin and eosin- (H&E-) stained slides, and diagnostic error was due to the lack of familiarity of nonspecialist surgical pathologists with rare and unusual neoplasms [6]. In their study, 80.4% cases were from community hospitals, 9.6% from academic medical centers and 5.6% from international hospitals, 4.2% from commercial laboratories, and less than 1% from government institutions.

Lehnhardt et al., in their review of 603 patients with soft tissue neoplasms received for second opinion over seven years, analyzed the types of lesions presenting challenges, discrepancies in grading, and impact of specimen type and the referring institute on the accuracy of the original diagnosis [7]. Their most frequent second-opinion diagnosis was liposarcoma, malignant fibrous histiocytoma (MFH)/sarcoma (NOS), synovial sarcoma, and leiomyosarcoma. They reported an overall discrepancy rate of 38.3%. They had the lowest agreement in cases of leiomyosarcoma at 25.8% and MPNST at 21.6%, which was not shared in this study (Table 1). In their study, in 1.4% of cases, the diagnosis was changed from malignant to benign and in 7.6% from benign to malignant. They reported concordant primary diagnosis in 28.3% for pathologists in private clinics, 29.6% for hospital pathologists, 36.8% for university hospital pathologists, and 70.5% for their own department [7]. In this current study, the concordance rate was 60% for cases from academic/university teaching hospitals and 60% from private clinics, compared with 54% for district general hospitals. This could be explained from the current practices in the UK, where more complicated cases are usually managed in larger referral centers, which are also university teaching hospitals, and many of these have a pathologist with special interest in soft tissue pathology. This is usually not the case with the district general hospitals, which could explain the lower concordance rate.

Al-Ibraheemi and Folpe more recently analyzed second-opinion discrepancy rates for pediatric bone and soft tissue neoplasms. There was agreement in 71% of cases, while 21% had minor and 8% had major discrepancies [8]. Their study did not include the cases of undifferentiated neoplasms reported in this audit, reflecting higher incidences of such tumors in the adult population (Table 1).

In a more recent study from France, Perrier et al. reported a discrepancy rate of 14%, with higher probability of discordance for a final diagnosis of desmoid tumors in comparison to liposarcomas [9]. They performed a cost analysis and found that centralized histologic reviews are likely to provide cost savings compared to the cost of additional treatment in case of a wrong diagnosis. With the discordance rate of 38% in this study and major diagnostic errors in 148 cases, a wrong diagnosis means unnecessary treatment to the patient with additional financial burdens on the National Health Service.

There were insufficient cases in this study to analyze grading differences, particularly because the focus of a second-opinion consultation was for diagnosis rather than for reporting prognostic factors such as grading. Grading was not applicable in 465 cases (80%) as they were benign or intermediate soft tissue neoplasms, nonmesenchymal tumors, and undifferentiated malignant neoplasms or showed discrepancy in diagnosis between the original diagnosis and the expert second opinion, making grading inappropriate. Although grading discrepancy assessment would have been relevant in 116 cases (20%) of malignant soft tissue tumors, many sarcomas types are associated with aggressive behavior, such that grading was not mentioned in the original or second-opinion report. Grading was given (in the original or second opinion report) in 26 cases (5%). Of these, the grading matched only in nine cases and was not mentioned in the original report in 13 or in the second-opinion report in four.

This study included 20 melanocytic lesions, 14 mesotheliomas, and 33 carcinomas amongst other nonmesenchymal lesions in the second-opinion diagnosis (Table 1). Of the nine cases of melanoma/clear cell sarcoma with diagnostic discrepancy, one case had an original diagnosis of Spitz nevus, one was of leiomyosarcoma, and in seven cases a wide range of differentials was offered as the original diagnosis. Amongst seven cases of mesothelioma with diagnostic discrepancy, four had an original diagnosis of carcinoma, two of poorly differentiated small cell sarcoma, and one case from the pleura had a diagnosis of synovial sarcoma. In 15 cases where a second opinion of poorly differentiated carcinoma was given, 13 were initially diagnosed as mesenchymal neoplasms, one as a poorly differentiated epithelioid neoplasm, and one as epithelioid hemangioendothelioma. For certain lesions (e.g., pericytic tumors, myoepithelial tumors, angiomatoid fibrous histiocytoma, acral fibromyxoma, and genital stromal mesenchymal tumors), the high level of diagnostic discrepancy could be due to the lack of familiarity of general pathologists for these neoplasms, as well as their overlapping morphology with other tumors. The 24 miscellaneous benign nonneoplastic cases (4%) on second opinion included organizing hematoma (2), scar tissue (1), fibrosis (5), fat necrosis (1), granulation tissue (5), cutaneous ossification (1), benign cyst (2), and five cases for which it was uncertain as to whether a specific lesion was represented. Only two of this group showed diagnostic discrepancies. A case of Wegener's granulomatosis was diagnosed as Kimura disease, and another case of possible adenomatoid tumor was diagnosed as reactive mesothelial proliferation on second opinion.

Referral cases are those referred to the tertiary center for further clinical management. These require mandatory review of the original histology and hence, are typically not as diagnostically challenging as second opinions. In 2009, we assessed diagnostic discrepancies for referred cases from 349 specimens over one year and found a total discrepancy rate of 26.6% including 93 cases of which 38 (11%) were major and 55 (15.6%) were minor discrepancies [4]. The largest group of discrepancy in 2009 was malignant to malignant diagnoses (15 cases) and the most discrepant diagnosis was for gastrointestinal stromal tumor (GIST) (seven cases), with the main reason being the misinterpretation of CD117 immunohistochemistry. The next groups of major discrepancy were smooth muscle and adipocytic tumors and then grading discrepancies. 203 (58%) were from district general (community) hospitals, 120 (34%) were from teaching hospitals, and 26 (8%) were from overseas university hospitals. In 2014, a similar study assessed 350 referral specimens over one year [5]. There was diagnostic agreement of 71.8%, with minor discrepancy rate of 11.8%, including grading discrepancies and 16.4% major discrepancies. There were seven cases of GIST and 19 smooth muscle tumors with discrepancies, although only five adipocytic tumors had diagnostic discrepancy. 230 (66%) were from district general hospitals, 83 (24%) were from teaching hospitals, 8 (2%) were from private laboratories, and 27 (8%) were from overseas.

The numbers of second opinion cases for the one-year period seen in this study are significantly greater than the numbers seen in the previous referral case audits (Table 10). This would reflect the increasing overall workload at this tertiary center and possibly also might be due to increasing subspecialization of pathologists, leading to more requests for second-opinion diagnoses for putative soft tissue tumors. There was overall diagnostic agreement of 73% and 71.8% in the previous referral studies compared to 62% in this audit, in line with the greater diagnostic complexity of second-opinion cases. This comprised a large group of 89 cases where it was difficult even to determine the tumor lineage, and these were often described as undifferentiated malignant neoplasms or classified by their predominant morphologic features, for example, undifferentiated pleomorphic, spindle cell, or small-cell sarcomas. The majority of cases (49%) in this study were from teaching hospitals and 42% from community hospitals (Table 9). This might be due to more complex cases being treated by larger teaching hospitals in the UK. In all these studies, there was no correlation between the incidence of discrepancy and the type of referring institution. In this study, 148 cases (25%) were termed major discrepancies and included discrepancies where tumors were placed in different categories of benign/intermediate/malignant behavior, considerably changing management (Tables 2–8). This higher percentage of major discrepancies in this audit, compared to 11 and 16.4% in the previous audits for referral cases, emphasizes the importance of obtaining expert second opinion for soft tissue neoplasms. 40 cases (7%) were considered as minor discrepancies leading to minor management change, while for 33 cases (5%), although there was a change in the diagnosis, this did not impact further management.

There were nine diagnoses of GIST in this study after second opinion (1.5%), and diagnostic discrepancy was noted in four; in these, the diagnosis was changed from mesothelioma (on morphology), SFT (DOG1 positive but no *KIT* or *PDGFRA* mutations on second-opinion workup), “vascular lesion” (DOG1 negative, *PDGFRA* mutation positive), and leiomyoma (*KIT* mutation positive). This indicates that although GIST is one of the commonly seen tumors in referral practice, it is not often sent for second opinion; these do not seem to pose a diagnostic challenge because of the awareness for GIST amongst general pathologists. Similarly, in this study, smooth muscle tumors also showed a lower discrepancy rate when compared to the fibrohistiocytic and adipocytic tumors. Although the fibroblastic/myofibroblastic tumors formed the largest group of tumors sent for second opinion, they overall showed a lower discrepancy rate when compared to other groups of tumors.

In terms of the utility of immunohistochemistry in second-opinion diagnoses, spindle cell neoplasms were found to be particularly challenging due to the overlap in their morphologic features, and the fact that some sarcomas such as low-grade fibromyxoid sarcoma (LGFMS) have typically bland cell morphology and can be missed if diagnostic awareness is lacking. MUC4 was performed in 20 myxoid spindle cell neoplasms, of which for six this helped towards the diagnosis of LGFMS. Of 13 cases of fibromatosis, seven were confirmed with positive *β*-catenin immunohistochemistry. Conversely, fibromatosis can be overcalled, as *β*-catenin can be focally positive in scar tissue, which was seen in one case. Amongst nine cases of synovial sarcoma, TLE1 was positive in seven. TLE1 was used in 14 other cases and found to be focally positive in other tumors including MPNST, carcinoma, and SFT. In two cases of undifferentiated carcinoma, INI1 was positive and useful in excluding epithelioid sarcoma, whilst in two cases the absence of nuclear INI1 was used to confirm the diagnosis of epithelioid sarcoma. Nevertheless, most diagnoses were overturned based on morphologic grounds alone.

In terms of the utility of molecular genetic testing, this was performed in 190 cases (32%), with test selection dependent on tumor morphology and immunophenotype. For the large group of undifferentiated malignant neoplasms described earlier (Table 1), a wider panel of molecular testing was used to exclude specific entities before putting them under the category of undifferentiated neoplasms. For example, in 16 such cases, RT-PCR for *EWSR1* fusion transcripts was performed in 10, fluorescence *in situ* hybridization (FISH) for *MDM2* amplification in four, molecular investigations for *SS18-SSX1/2* translocations in four, *PAX-3/7-FOXO1* translocations in two, *BRAF* and *KIT* mutational analysis in two, and *PDGFRA* mutational analysis in one.

Amongst tumors with predominant spindle cell morphology, molecular testing was used in 62 cases. The various tests were for exclusion of myxoid liposarcoma with *FUS-DDIT3* fusion transcripts with RT-PCR (one case), *MDM2* gene amplification with FISH (negative in all three cases), *FUS-CREB3L1/2* fusion transcripts with RT-PCR (13 cases, for which diagnosis of LGFMS was confirmed in four), various *EWSR1* fusion transcripts with RT-PCR (negative in all 11 cases), *ALK* gene rearrangements (11 cases, with confirmation of a diagnosis of inflammatory myofibroblastic tumor (IMT) in three), *KIT* mutational analysis (13 cases of which GIST was confirmed in four), *PDGFRA* mutational analysis (10 cases of which GIST was confirmed in one), *JAZF1-SUZ12* fusion transcripts (negative in all three cases), and *SS18-SSX1/2* fusion transcripts (17 cases). Of the latter, seven cases of synovial sarcoma with spindle cell morphology were confirmed with positive molecular results. Another well-documented diagnostically challenging entity is nodular fasciitis which often leads to confusion with sarcoma and other myofibroblastic proliferations, especially as immunohistochemistry (typically SMA expression alone) is not always helpful. We had two such cases for which the original diagnosis was low-grade spindle cell tumor and atypical nodular spindle cell lesion, with review diagnosis changed to nodular fasciitis based on morphologic appearances. Previous studies have shown that for difficult cases, FISH analysis for *USP6* gene rearrangement is useful, with detection in 74.4% [15]. One case of ossifying fasciitis was positive for *USP6* gene fusion with FISH in our study. A case of spindle cell tumor NOS was confirmed as angiomatoid fibrous histiocytoma due to the finding of *EWSR1-CREB1* fusion transcripts with RT-PCR.

In tumors with small round cell morphology, amongst nine cases of rhabdomyosarcoma, in eight, RT-PCR for *PAX3/7-FOXO1* fusion transcripts was performed and was positive in one case, diagnosed as alveolar rhabdomyosarcoma. Three cases of Ewing sarcoma were confirmed by the finding of *EWSR1-FLI1* fusion transcripts with RT-PCR. In two cases of poorly differentiated synovial sarcoma with round cell morphology, the diagnosis was made based on positive *SS18-SSX1/2* fusion transcripts, and two cases were also positive for TLE1.

Adipocytic neoplasms represent one of the most diagnostic challenging soft tissue tumors for general pathologists. FISH for *MDM2* amplification status was performed in 61/74 cases of adipocytic neoplasms and was found to be the most useful technique for confirming or excluding a diagnosis of well-differentiated liposarcoma (WDL) or dedifferentiated liposarcoma (DDL). This test failed in three cases. Nine cases of lipoma were received with a request to exclude WDL, of which FISH for *MDM2* amplification was performed in eight and found to be negative. One spindle cell lipoma was diagnosed on morphologic grounds alone. In 26 cases, *MDM2* FISH helped to confirm the diagnosis of liposarcoma, with 16 WDL and 10 cases of DDL, all of which were intra-abdominal. Of the four tumors originally thought to be lipomas which turned out to be WDLs with FISH, for two cases the original diagnosis was of pleomorphic lipoma, highlighting the difficulty in interpretation of pleomorphic floret type-cells. Of the seven cases initially diagnosed as WDL which turned out to be benign, three were lipomas with fat necrosis, with inflammatory cells and fibrosis surrounding fat necrosis being misinterpreted as atypical stromal cells and the fibrous septa of WDL. As differentiated adipocytic lesions can pose a diagnostic challenge even to specialist soft tissue pathologists and *MDM2* amplification with FISH represents the diagnostic gold standard in distinguishing WDL from benign adipocytic lesions [16–18], there should be a low threshold for performing FISH for *MDM2* amplification. One case was originally reported as nondiagnostic then diagnosed as DDL based on the finding of *MDM2* amplification with FISH on second opinion. RT-PCR for *FUS-DDIT3* gene fusion was performed in one case of unclassifiable adipocytic tumor and in two cases of lipoblastoma. In 18 cases, CDK4 immunohistochemistry was also performed and correlated with *MDM2* FISH results in 16 but was contradictory in two. p16 was performed in five cases along with CDK4, but CDK4 was more useful in adipocytic tumors as p16 can also be positive in areas of fat necrosis.

Molecular diagnosis was utilized in 14 cases initially diagnosed as melanoma. Two cases were received with request for *BRAF* mutational analysis, which was detected in one case. In seven, analysis for *EWSR1-CREB1* and *EWSR1-ATF1* fusion transcripts or *EWSR1* gene rearrangement was performed to exclude clear cell sarcoma (CCS). Two diagnoses were changed to CCS based on positive molecular findings, while in the remaining five cases mutational analysis for *BRAF* was performed, with confirmation as melanoma in three where the mutation was detected. In one case, the material was insufficient, and the other case was still diagnosed as melanoma morphologically. In one case of malignant PEComa, *BRAF* and *KIT* mutations were found to be negative, with melanoma excluded. In 6 cases of carcinomas with sarcomatoid or epithelioid morphology, *SS18-SSX1/2* was useful in excluding synovial sarcoma. In three cases of mesothelioma, in two where the differential diagnosis was of synovial sarcoma, *SS18-SSX1/2* molecular analysis was performed, and in one case FISH for *ALK* gene rearrangement was performed to exclude IMT. In one case of chondroma, RT-PCR for *EWSR1-NR4A3* gene fusion was performed to exclude extraskeletal myxoid chondrosarcoma. In three cases of myoepithelioma, FISH for *EWSR1* gene rearrangement was performed, but was negative in all, and the diagnosis was made on morphology alone.

As it is evident from the discussion, diagnosis in most consultation cases was largely done based on morphology alone by specialist soft tissue pathologists, with subsequent selection of an appropriate confirmatory immunopanel, and ancillary molecular investigations were often as useful in the exclusion of specific diagnoses as they were in confirmation. Confirmatory molecular investigations for specific neoplasms are important for the use of targeted therapies such as BRAF or CDK4 inhibitors (in confirming that patients have melanoma rather than CCS and for patients with DDL, respectively) and for patient enrolment into appropriate clinical trials. Furthermore, there is increasing recognition of the importance of histology-tailored therapy in treating soft tissue sarcomas, making accurate histologic diagnosis fundamental to treatment selection [19]. This has been highlighted by the introduction of tyrosine kinase inhibitors in GIST, including imatinib, sunitinib, and regorafenib. However, certain GIST mutations are known to be resistant to these agents (*PDGFRA* D842V), and there are new agents available in clinical trials that have shown efficacy against these treatment-resistant subtypes [20]. This highlights the importance of expert multidisciplinary care for patients with rare cancers. In addition, there are a number of ongoing subtype-specific trials in various sarcoma subtypes including epithelioid sarcoma, angiosarcoma, undifferentiated pleomorphic sarcoma, and liposarcoma. Accurate histopathological diagnosis is also of critical importance not only for discussing treatment options but also for prognosis with patients and their families.

In summary, the diagnostic discrepancy rate of 38% for second-opinion cases highlights the importance of obtaining opinions from specialist soft tissue pathologists in challenging cases, especially when working in centers where newer antibodies and molecular testing are not available. Obtaining an accurate diagnosis by second opinion is essential due to the diagnostic implications on management.

## Figures and Tables

**Table 1:** Summary of second-opinion cases received according to assigned referral and second-opinion lineage/differentiation.

Tumor categories with second-opinion diagnoses	Total no. of cases	No discrepancy in opinion	Discrepancy in opinion	% with discrepancy
*Specific lineage assignable*				
Fibroblastic/myofibroblastic	99	65	34	34%
Adipocytic	74	42	32	43%
Smooth muscle	35	25	10	28%
Vascular	32	22	10	31%
Nerve sheath	32	21	11	34%
Skeletal muscle	14	12	2	14%
So-called “fibrohistiocytic”	25	9	16	64%
Pericytic	3	1	2	67%

*Lesions of uncertain differentiation*				
Synovial sarcoma	9	6	3	33%
Atypical fibroxanthoma	7	5	2	28%
Pleomorphic hyalinizing angiectatic tumor	2	2	0	—
Myoepithelial tumors	9	6	3	33%
Ossifying fibromyxoid tumor	4	3	1	25%
Angiomatoid fibrous histiocytoma	1	0	1	100%
Intramuscular myxoma	1	0	1	100%
Acral fibromyxoma	1	0	1	100%
Epithelioid sarcoma	3	1	2	67%
*Undifferentiated pleomorphic sarcoma*	31	16	15	48%
*Spindle cell sarcoma (including postradiation sarcoma)*	27	13	14	52%
*Undifferentiated round cell tumor*	9	5	4	44%
*Undifferentiated malignant neoplasm*	22	16	6	27%
*Bone and cartilage tumors/lesions*	7	4	3	43%

*Others*				
Follicular dendritic sarcoma	2	2	0	—
Phyllodes tumor	3	3	0	—
Gastrointestinal stromal tumor	9	5	4	44%
Specialised genital stromal tumors	12	4	8	66%
Olfactory neuroblastoma	2	2	0	—
Ewing sarcoma	3	3	0	—
Melanoma, including clear cell sarcoma	20	11	9	45%
PEComa, including lymphangioleiomyomatosis	3	3	0	—
Meningioma	1	0	1	100%
*Mesothelioma*	14	7	7	50%
*Carcinoma*	33	18	15	45%
*Germ cell/sex-cord stromal tumors*	8	6	2	25%
*Miscellaneous benign lesions*	24 = 134	22 = 86	2 = 48	8%
Total cases	581	360	221	38%

**Table 2:** Fibroblastic/myofibroblastic tumors with discrepancy in opinion.

Original diagnosis	Second-opinion diagnosis	Comments
*Malignant to malignant*, *2 cases*		
Pleomorphic sarcoma	Myxoinflammatory fibroblastic sarcoma	
Low-grade spindle cell neoplasm	Low-grade fibromyxoid sarcoma/sclerosing epithelioid fibrosarcoma	MUC4 negative but FUS-CREB gene fusion present

*Malignant to Intermediate*, *6 cases*		
Low-grade fibromyxoid sarcoma	Fibromatosis	MUC4 negative and *β*-catenin positive
Fibrosarcoma	Fibromatosis	MUC4 negative and *β*-catenin positive
Gastrointestinal stromal tumor	Fibromatosis	KIT and PDGFRA mutational analysis negative and *β*-catenin positive
Synovial sarcoma	Inflammatory myofibroblastic tumor	ALK gene rearrangements absent
Sarcoma	Myxoinflammatory fibroblastic sarcoma	
High-grade sarcoma, possibly gastrointestinal stromal tumor	Solitary fibrous tumor	KIT and PDGFRA mutational analysis negative and focal TLE1 positive

*Malignant to benign*, *2 cases*		
Low-grade spindle cell tumor	Nodular fasciitis	
Leiomyosarcoma	Mammary-type myofibroblastoma	

*Benign to benign*, *13 cases*		
Fibrous hamartoma of infancy	Nodular fasciitis	
Spindle cell tumor	Fibroblastic tumor without malignant features	ETV6 negative, t(8; 15) present
Genital stromal fibroepithelial polyp	Superficial myofibroblastoma	
Benign fibrous histiocytoma	Superficial myofibroblastoma	
Inflammatory myofibroblastic tumor-type lesion	Fibroblastic proliferation of unknown significance	ALK and MUC4 immunostains negative
Lipoma	Intravascular fasciitis	CDK4 immunostain negative
Angiomyxoma	Fibroma	
Benign lesion	Myofibroblastoma	
Specialised genital stromal tumor	Myofibroblastoma	
Neurothekeoma	Fibromyxoma	MUC4 negative
Benign lesion	Pleomorphic fibroma	
Nodular fasciitis	Myofibroma	
Benign lesion	Fibroma	

*Benign to malignant*, *1 case*		
Proliferative myositis	Possibility of low-grade fibromyxoid sarcoma	

*Benign to intermediate*, *2 cases*		
Angiofibroma	Solitary fibrous tumor	
Benign spindle cell lesion	Solitary fibrous tumor	
*Intermediate to intermediate*, *1 case*		
Spindle cell neoplasm of uncertain malignant potential	Fibromatosis	

*Intermediate to benign*, *5 cases*		
Fibromatosis	Scar tissue	Focal *β*-catenin positive
Palmar fibromatosis	Ossifying fasciitis	USP6 positive
Atypical nodular spindle cell lesion	Nodular fasciitis	
Fibrous pseudotumor/solitary fibrous tumor	Fibroma of testis	
Fibromatosis	Proliferative myositis	

*Intermediate to malignant*, *2 cases*		
Solitary fibrous tumor	Low-grade fibromyxoid sarcoma	MUC4 positive and FUS-CREB gene fusion present
Dermatofibrosarcoma protuberans/solitary fibrous tumor	Myxofibrosarcoma	

**Table 3:** Adipocytic tumors with discrepancy in opinion.

Original diagnosis	Second-opinion diagnosis	Comments
*Malignant to malignant*, *12 cases*		
Malignant peripheral nerve sheath tumor	Dedifferentiated liposarcoma	MDM2 amplified
Atypical neoplasm	Well-differentiated liposarcoma	MDM2 amplified
Myxoid sarcoma	Dedifferentiated liposarcoma	MDM2 failed
Pleomorphic leiomyosarcoma	Dedifferentiated liposarcoma	MDM2 amplified
Leiomyosarcoma	Dedifferentiated liposarcoma	MDM2 amplified
Sarcomatoid renal cell carcinoma	Dedifferentiated liposarcoma	
Pleomorphic sarcoma	Dedifferentiated liposarcoma	
Sarcoma	Dedifferentiated liposarcoma with rhabdomyosarcomatous areas	MDM2 amplified
Leiomyosarcoma	Dedifferentiated liposarcoma	MDM2 amplified
Undifferentiated sarcoma	Dedifferentiated liposarcoma	MDM2 amplified, ALK negative, CDK4 focal, INI1 +

Low-grade sarcoma	Dedifferentiated liposarcoma	
Undifferentiated sarcoma	Well-differentiated liposarcoma	MDM2 amplified

*Malignant to benign*, *7 cases*		
7 cases of liposarcoma	Lipoma with or without fat necrosis	MDM2 not amplified in all cases, but CDK4 positive in 1 case

*Benign to benign*, *5 cases*		
Spindle cell lipoma	Fibrolipoma	MDM2 not amplified
Hamartoma	Fibrolipoma	
Spindle cell lipoma	Lipoblastoma	
Fibrous hamartoma of infancy	Lipoblastomatosis	
Myxoma	Spindle cell lipoma	

*Benign to malignant*, *5 cases*		
4 lipomas, 1 stromal lesion	Well-differentiated liposarcoma	MDM2 amplified in all cases

*Intermediate to malignant*, *2 cases*		
Inflammatory myofibroblastic tumor	Dedifferentiated liposarcoma	MDM2 amplified
Solitary fibrous tumor	Dedifferentiated liposarcoma	MDM2 amplified, CDK4 positive, MUC4, DOG1, ALK negative
*Nondiagnostic material*, *1 case*	Dedifferentiated liposarcoma	MDM2 amplified

**Table 4:** Smooth muscle tumors with discrepancy in opinion.

Original diagnosis	Second-opinion diagnosis
*Malignant to malignant*, *5 cases*	
Epithelioid rhabdomyosarcoma	Epithelioid leiomyosarcoma
Malignant spindle cell tumor	Cutaneous leiomyosarcoma
Myxofibrosarcoma	Leiomyosarcoma
Gastrointestinal stromal tumor	Leiomyosarcoma
Angiosarcoma	Leiomyosarcoma

*Benign to malignant*, *1 case*	
Cellular leiomyoma	Low-grade leiomyosarcoma

*Intermediate to malignant, 4 cases*	
Smooth muscle tumor of uncertain malignant potential	Low-grade leiomyosarcoma
Atypical leiomyoma	Low-grade leiomyosarcoma
Smooth muscle tumor of uncertain malignant potential	Leiomyosarcoma, grade 2
Smooth muscle tumor of uncertain malignant potential	Leiomyosarcoma

**Table 5:** Vascular tumors with discrepancy in opinion.

Original diagnosis	Second-opinion diagnosis
*Malignant to malignant*, *3 cases*	
Poorly differentiated carcinoma	Epithelioid angiosarcoma
Poorly differentiated malignant neoplasm	Epithelioid angiosarcoma
Gastrointestinal stromal tumor	Angiosarcoma

*Malignant to benign*, *1 case*	
Low-grade vascular lesion	Hemorrhage with hemangioma

*Malignant to intermediate*, *1 case*	
Metastasis of adenocarcinoma	Epithelioid hemangioendothelioma

*Benign to benign*, *2 cases*	
Myofibroma	Hemangioma
Angiolipomatous tumor	Hemangioma

*Benign to malignant*, *1 case*	
Epithelioid hemangioma	Low-grade angiosarcoma
*Intermediate to benign*, *2 cases*	

Kaposiform hemangioendothelioma	Hemangioma
Atypical vascularised tumor of unknown malignant potential	Vascular proliferation and sclerosis

**Table 6:** Nerve sheath tumors with discrepancy in opinion.

Original diagnosis	Second-opinion diagnosis
*Malignant to malignant*, *1 case*	

Melanoma or less likely cellular schwannoma	Malignant peripheral nerve sheath tumor

*Malignant to benign*, *3 cases*	

Low-grade sarcoma	Schwannoma

Low-grade spindle cell neoplasm	Schwannoma/neurofibroma

Spindle cell liposarcoma	Neurofibroma

*Benign to benign*, *1 case*	

Neural tumor	Plexiform cellular schwannoma

*Benign to intermediate*, *2 cases*	

Spindle cell lesion	Myxoid atypical neurofibroma

Granular cell tumor	Atypical granular cell tumor

*Benign to malignant*, *3 cases*	

Schwannoma	Malignant schwannoma

Benign neural tumor	Low-grade MPNST

Granular cell tumor	Malignant granular cell tumor

*Intermediate to malignant*, *1 case*	

Fibromatosis	Low-grade MPNST

MPNST: malignant peripheral nerve sheath tumor.

**Table 7:** Fibrohistiocytic tumors with discrepancy in opinion.

Original diagnosis	Second-opinion diagnosis
*Malignant to benign*, *3 cases*	

Malignant spindle cell tumor	Giant cell tumor of soft parts

Leiomyosarcoma	Fibrous histiocytoma

Vascular lesion with malignant features	Epithelioid fibrous histiocytoma

*Benign to benign*, *8 cases*	

Clear cell soft tissue neoplasm	Pigmented villonodular synovitis

Benign fibrous histiocytoma	Xanthogranuloma

Neurothekeoma	Benign fibrous histiocytoma

Nodular fasciitis	Fibrous histiocytoma

Infantile myofibromatosis	Juvenile xanthogranuloma

Vascular lesion, favouring hemangioma	Juvenile xanthogranuloma

Mesenchymal neoplasm, likely vascular	Aneurysmal fibrous histiocytoma

Vascular lesion	Benign fibrous histiocytoma/capillary hemangioma

*Benign to intermediate*, *1 case*	

Dermatofibroma	Dermatofibrosarcoma protuberans

*Intermediate to benign*, *3 cases*	

Dermatofibrosarcoma protuberans	Deep dermatofibroma

Dermatofibrosarcoma protuberans	Cellular dermatofibroma
Smooth muscle tumor of uncertain malignant potential	Cellular fibrous histiocytoma

**Table 8:** Changes in assignment of tumor behavior.

Category	Number of cases	Major discrepancy	Minor discrepancy	No impact on management/not comparable
Malignant to malignant	99	75	18	6
Malignant to benign	12	11	1	
Benign to malignant	20	20		
Malignant to intermediate	11	11		
Benign to benign	39	1	12	26
Benign to intermediate	6	6		
Intermediate to benign	18	12	5	1
Intermediate to malignant	15	11	4	
Intermediate to intermediate	1	1		
Total	221	148	40	33

**Table 9:** Percentage of diagnostic concordance according to type of referring institution.

Type of hospital	Number of cases	% of concordance
Academic/teaching centers	285 (49%)	173 cases (60%)
Community (district general) hospitals	242 (42%)	131 cases (54%)
Private institutions	38 (6%)	23 cases (60%)
Overseas	16 (3%)	11 cases (69%)

**Table 10:** Tumor groups causing major differences in opinion/difficulties between referral and consultation opinion.

	2009	2014	2012 (current)
Type of cases	Referral	Referral	Second opinion (consultation)
Number of specimens	349	350	581
% of discrepancy	26.6%	28.2%	38%
Major	11%	16.4%	25%
Minor	15.6%	11.8%	7% (Table 8)
Major problem areas identified	Diagnosis of GIST and smooth muscle tumors	Diagnosis of smooth muscle tumors and GIST	Diagnosis of undifferentiated neoplasms, fibrohistiocytic and adipocytic tumors

## Data Availability

Data are available upon request from the corresponding author.
